# Challenges in Forecasting Antimicrobial Resistance (Response)

**DOI:** 10.3201/eid2907.230617

**Published:** 2023-07

**Authors:** Sen Pei

**Affiliations:** Columbia University, New York, New York, USA

**Keywords:** health-associated infection, real-time forecasting, antimicrobial resistance, bacteria, United States

**In Response:** Real-time evaluation of predictive models for antimicrobial resistance (AMR) is critical for real-world applications, as indicated in our recently published article (*1*). Aldeyab and Lattyak introduced a threshold-logistic regression model that links antimicrobial drug use to AMR prevalence in hospital settings (*2*). The authors advocate implementing and testing this model in hospitals to assess operational utility. I agree that this is a practical starting point to challenge time-series model use for real-time AMR predictions. Most time-series models have been validated in retrospective analyses. Translational research is needed to promote the use of those models for real-world AMR control.

The authors mention several practical considerations when applying time-series models in real time, including stationarity of both predictor and target variables and criteria for model recalibration. Evaluating methods to address those issues is crucial to achieve desirable performance in hospital settings. In addition to those technical challenges, several broader questions remain regarding model design and utility. First, how much AMR prevalence variation can be explained by antimicrobial drug use? Are there other essential factors (e.g., community introduction) that should be included in the model? Second, how will healthcare providers and hospitals use AMR forecasts? What policies will be informed by forecasts, and what are the downstream effects? Answers to those questions will help determine the eventual real-world utility of predictive models.

Evaluating real-time AMR prediction is a complicated task. By drawing experience from computer vision (*3*) and forecasts for other infectious diseases (*4*–*6*), open-access challenges with transparent and fair evaluation methods run in a common task framework (*7*) can substantially stimulate the advance of predictive methods and might produce robust application models. Such collaborative efforts are needed to evaluate existing methods, identify difficulties and solutions, and push the operational use of AMR predictive models forward.
